# A Comprehensive Literature Review of Treatment-Emergent Integrase Resistance with Dolutegravir-Based Regimens in Real-World Settings

**DOI:** 10.3390/v15122426

**Published:** 2023-12-14

**Authors:** Cassidy Henegar, Emilio Letang, Ruolan Wang, Charles Hicks, Dainielle Fox, Bryn Jones, Annemiek de Ruiter, Vani Vannappagari

**Affiliations:** 1 ViiV Healthcare, 406 Blackwell Street, Suite 300, Durham, NC 27701, USA; ruolan.h.wang@viivhealthcare.com (R.W.); charles.x.hicks@viivhealthcare.com (C.H.); dainielle.x.fox@viivhealthcare.com (D.F.); vani.x.vannappagari@viivhealthcare.com (V.V.); 2 ViiV Healthcare, P.T.M., Severo Ochoa 2, 28760 Tres Cantos, Madrid, Spain; 3 ViiV Healthcare, 980 Great West Road, Brentford TW8 9GS, Middlesex, UK

**Keywords:** dolutegravir, integrase inhibitor, real-world, resistance-associated mutation

## Abstract

After a decade of dolutegravir (DTG) use in various antiretroviral therapy combinations and in diverse populations globally, it is critical to identify HIV strains with reduced drug susceptibility and monitor emergent resistance in people living with HIV who experience virologic failure while on DTG-based regimens. We searched the PubMed, Embase, and Cochrane databases to identify studies that reported DTG resistance-associated mutations (RAMs) emerging under selection pressure. Our review showed that RAMs conferring resistance to DTG were rare in 2-drug and 3-drug regimens used in real-world cohorts, corroborating data from clinical trials. The potency of DTG in maintaining virologic suppression was demonstrated, even in cases of pre-existing resistance to companion drugs in the regimen. Estimates of DTG RAMs depended on the population and certain risk factors, including monotherapy, baseline resistance or lack of genotypic testing, treatment history and prior virologic failure, and suboptimal treatment adherence. The RAMs detected after virologic failure, often in heavily treatment-experienced individuals with prior exposure to integrase strand transfer inhibitors, were G118R, E138K, G140A/C/R/S, Q148H/K/R, N155H, and R263K. Overall, these data highlight the durable effectiveness and high barrier to resistance of DTG as part of combination antiretroviral therapy in a wide variety of settings.

## 1. Introduction

Dolutegravir (DTG) is a second-generation integrase strand transfer inhibitor (INSTI) with a high barrier to resistance, high potency in the absence of pharmacokinetic boosters, and substantial clinical data supporting its use in 2- and 3-drug regimens for treatment-naive and treatment-experienced people living with HIV [1,2,3,4,5]. Globally, HIV treatment guidelines recommend DTG-based antiretroviral therapy (ART) for first-line treatment and as a switch option for virologically suppressed people [1,3,6]. Over the decade since its approval, DTG has become widely available, and public health systems in many countries have initiated rollout programs for DTG-based regimens in combination with other antiretroviral drugs (ARVs) [7]. With the availability of data from large observational cohort studies, it is now possible to assess the effectiveness of DTG as part of a variety of regimens prescribed for diverse populations in real-world clinical settings [5].

Regional surveillance studies of transmitted resistance generally report low prevalence of INSTI-associated drug resistance. One study of 2705 people diagnosed with HIV (from 2018–2021) across five European countries reported the prevalence of INSTI mutations to be 0.3% [8]. Another study of serum samples from 474 ART-naive people with HIV in China from 2018 to 2020 reported an INSTI resistance mutation prevalence of 0.63% [9]. In the United States, surveillance of 50,747 newly diagnosed individuals from 2014 to 2018 showed a prevalence of 0.8% for INSTI resistance-associated mutations (RAMs) [10]. Major mutations (occurring alone or in combination with accessory mutations) that confer high levels of resistance to multiple INSTIs include G118R, R263K, Q148H/K/R, G140A/C/S, and N155H [11]. Multiple mutations are needed to confer high-level resistance to DTG [12]. Considering the widespread use of second-generation INSTIs, the low prevalence of transmitted drug resistance demonstrates their high barrier to resistance, regardless of the other regimen components. Indeed, the prevalence of pre-treatment non-nucleoside reverse transcriptase inhibitor (NNRTI), nucleoside reverse transcriptase inhibitor (NRTI), and protease inhibitor (PI) mutations is generally higher at 12.9% (efavirenz or nevirapine), 5.4%, and 0.4% globally (2014–2020), respectively, and 12.0%, 6.9%, and 4.2% in the United States (2014–2018), respectively [10,13]. Furthermore, this informs the recommendations from guidelines to initiate treatment with a second-generation INSTI in cases where baseline resistance testing is not immediately available [1,3,6].

The efficacy of DTG as the first-line ART among treatment-naive people living with HIV and as a switch option among treatment-experienced individuals with a variety of treatment histories has been studied extensively in clinical trials. In the five industry-sponsored phase 3 studies of a combined 1386 treatment-naive participants who received DTG + 2 NRTIs (SPRING-2, *n* = 411 [14]; SINGLE, *n* = 414 [15]; FLAMINGO, *n* = 242 [16]; INSPIRING *n* = 69 [17]; ARIA, *n* = 250 [18]), there were no cases of treatment-emergent resistance (to any ARV class) through 24 to 144 weeks of follow-up. Through 144 weeks in the phase 3 GEMINI-1 and GEMINI-2 trials that included 1433 ART-naive individuals treated with DTG (716 with DTG + lamivudine [3TC] and 717 with DTG + tenofovir disoproxil fumarate/emtricitabine [TDF/FTC]), treatment-emergent NRTI (M184V, selective for 3TC) and INSTI (R263R/K) resistance was observed in a single individual taking DTG + 3TC, at weeks 132 and 144, respectively [19]. This emergent resistance was considered to be due to treatment non-adherence, and the participant withdrew from the study for lack of efficacy at week 148. Additionally, in the phase 3b STAT trial, in which 131 treatment-naive individuals initiated DTG/3TC treatment in a US test-and-treat setting before availability of resistance testing results, no treatment-emergent resistance was observed over 48 weeks [20]. Therefore, the incidence of treatment-emergent resistance in treatment-naive individuals in the industry-sponsored clinical program is 0.034% (1/2950) for any DTG-based regimen and 0.12% (1/847) for DTG + 3TC 2-drug regimens.

The efficacy of DTG-based ART has also been demonstrated in treatment-experienced populations, including individuals with prior virologic failure (VF) and those with multidrug-resistant HIV-1. In phase 3 clinical trials in which participants who were virologically suppressed on a 3- or 4-drug regimen with no history of treatment failure were switched to a DTG-based regimen, no treatment-emergent INSTI resistance was observed during the STRIIVING (DTG/3TC/abacavir [DTG/3TC/ABC], *n* = 553) [21], SWORD-1 and SWORD-2 (DTG + rilpivirine [DTG + RPV], *n* = 990) [22], TANGO (DTG/3TC, *n* = 369) [23], and SALSA (DTG/3TC, *n* = 246) [24] trials, with follow-up ranging from 24 to 144 weeks; of note, 6 SWORD-1/-2 participants had NNRTI- or RPV-associated resistance mutations at failure (1 had baseline RPV resistance). In a 10-year follow-up of the phase 3 SAILING trial, in INSTI-naive participants with resistance to ≥2 ARV classes, a subset of 29 participants who switched to DTG + 2 NRTIs and only ever received NRTIs as their background regimen had low rates of VF and integrase substitutions, with minimal effect on DTG susceptibility [25]. In the phase 3 VIKING-3 trial, participants with VF and INSTI-resistant virus received DTG 50 mg twice daily (replacing raltegravir [RAL] or elvitegravir [EVG] in their failing regimen); after background ART optimization at day 8, 69% achieved HIV-1 RNA <50 copies/mL at week 24 [26]. In the phase 3b DAWNING study, participants failing first-line therapy with an NNRTI + 2 NRTIs and who switched to DTG + 2 NRTIs had low VF rates overall (11/312; 3.5%). Of the 11 participants with VF, five (45.5%) had baseline M184V/I (selective for FTC and 3TC [XTC], used in their regimen); of these, one had emergent INSTI RAMs (H51H/Y, G118R, E138E/K, R263R/K) and one had emergent INSTI (G118R) and NRTI (D67N) RAMs [27]. However, overall virologic suppression rates were high (84% by Snapshot algorithm) regardless of whether participants had pre-existing NRTI RAMs. Additionally, a post hoc analysis from the phase 3 BRIGHTE study showed that inclusion of twice-daily DTG in optimized background therapy (OBT) with fostemsavir appeared to have the greatest effect on virologic outcomes in heavily treatment-experienced (HTE) individuals at week 96 compared with other ARVs [28]. Together, these data highlight DTG as a treatment option for achieving virologic suppression in treatment-experienced populations, even with previous VF.

Phase 3 clinical trials provide insight into the efficacy of a regimen under controlled conditions and with more oversight than in real-world clinical practice settings. HIV clinics may treat individuals from populations that are underrepresented in clinical trials, such as people aged ≥50 years and those who inject drugs [29,30]. Additionally, though observational data suggest that the infrequent incidence of mutations conferring resistance to second-generation INSTIs mirrors clinical trial data, use in real-world clinical settings may not always be consistent with on-label use. Real-world evidence can complement clinical trial data and show how treatment-emergent resistance develops across a broader population of people living with HIV and using DTG-based regimens in real-life settings. In this review, we identified and reviewed published or presented studies that reported data on treatment-emergent mutations conferring resistance to INSTIs in people living with HIV during treatment with DTG-based 2-drug or 3-drug regimens outside of clinical trial settings.

## 2. Methods

### 2.1. Literature Review

We performed a comprehensive search of the National Center for Biotechnology Information PubMed, Embase (ProQuest), and Cochrane databases for all English-language papers as well as databases and congress abstract books for all English-language congress presentation abstracts, using the search strings listed in Tables S1–S3, last dated 25 July 2023. A post hoc search was performed to include presentations from 2 additional congresses through 20 October 2023.

Papers and abstracts were reviewed in multiple stages. First, duplicate publications were excluded, and the remaining publications were filtered by relevant study type (real-world evidence study, case study, or systematic review/meta-analysis [inclusive of observational studies]). Case studies; systematic reviews; and cohort studies that did not identify risk factors for treatment-emergent resistance to DTG (such as reporting use of DTG monotherapy; lack of viral load testing; or individuals with prior VF, suboptimal adherence, or with extensive treatment experience) were excluded. The remaining studies were then included in subsequent rounds of review if they discussed drug resistance, then direct or likely INSTI RAMs, then confirmed/likely clinical failure of any INSTI regimen to treatment-emergent resistance mutations. Finally, publications were further filtered by confirmed resistance to DTG and INSTI RAMs. A manual post hoc search of congress material titles was performed during full-text screening to determine whether any content was published but not identified in the database search. Congress materials linked to published articles, whether identified in the database search or the manual post hoc search, were excluded in favor of peer-reviewed published articles. If multiple publications had potential overlap of cohort populations, outcomes from the study with the larger N and/or newer or more relevant data were used; however, all potential overlap may not have been identified.

### 2.2. Data Extraction

Data were extracted independently by 4 individuals using standardized spreadsheets for manuscripts and congress abstracts. Discrepancies were handled by jointly reviewing full-text articles, and studies deemed to be linked to one another (i.e., reporting on the same cohort) were flagged. Study characteristics included region/country, type, design, and setting; regimen type; treatment duration; and follow-up period. Cohort population information included total number of individuals included, number who were treatment-naive or treatment-experienced, and number with baseline resistance mutations. Information related to DTG treatment failure included number of “blips” on DTG-based regimens (single measures of 50–200 copies/mL), number of individuals with confirmed VF on DTG-based regimens, study definition of VF, rates of adherence, number of individuals with DTG-based treatment-emergent resistance mutations, type of mutation(s) present, and class of mutation resistance.

## 3. Results

### 3.1. Treatment-Emergent Resistance by Regimen

The proportions of people living with HIV who experienced VF on DTG-based therapy and developed treatment-emergent integrase mutations by study and regimen are shown in Table 1. While the definition of VF varied, many included 1 or 2 consecutive measurements of “detectable” HIV-1 RNA [31,32], or 1 or 2 consecutive measurements of HIV-1 RNA > or ≥50 copies/mL [33,34,35,36,37,38,39,40,41], >200 copies/mL [40,42,43], >400 copies/mL [37,44], ≥500 copies/mL [41], and/or >1000 copies/mL [45,46] as part of study-defined VF criteria. Many did not explicitly define VF criteria [47,48,49,50,51,52,53,54,55,56,57,58,59,60,61,62]. Real-world data by regimen are described in further detail in the following sections.

#### 3.1.1. Dolutegravir/Lamivudine/Abacavir (DTG/3TC/ABC)

Two large cohort studies described VF and emergent resistance with DTG/3TC/ABC. One multicenter study in France evaluated RAMs in people living with HIV and on first- or second-line INSTI-based ART from 2019 to 2022 [33]. Of the 3219 individuals receiving DTG-based regimens in the analysis, 179 (5.6%) experienced VF and 24 (0.75%) had treatment-emergent INSTI RAMs (DTG/3TC/ABC, 11/1709 [0.64%]; DTG + 3TC, 6/644 [0.93%]; DTG + RPV, 7/866 [0.81%]): G140S, Q148H, E92K, and N155H. Additionally, 7 individuals failing first-line DTG-based regimens had emergent M184V/I mutations and 14 failing second-line DTG-based regimens had emergent NRTI (*n* = 7, M184V) and NNRTI (*n* = 3, E138A; *n* = 1 each, M230L, K101E, Y181C, V179L) mutations at VF.

In a Canadian database study of 928 individuals who started INSTI-based regimens between 2012 and 2014, 392 were on a DTG-based regimen with a 3TC + ABC or TDF + FTC/3TC backbone. During the first year of follow-up, 65 (16.6%) were “not suppressed” (rebound after suppression, lack of suppression within 6 months, or consecutive blips); of these, 3 (4.6%) were receiving DTG + 3TC + ABC and developed emergent INSTI mutations (*n* = 1, T66I; *n* = 2, R263K). Emergent NRTI mutations (M184V/I) were detected in 4 individuals, including 2 with R263K mutations. In this study, suboptimal adherence (defined as <80% adherence to ART regimen) was associated with a significant (2.5-fold) likelihood of developing emergent resistance [43]. Emergent drug resistance was detected in people with an apparent ≥95% ART prescription fill history, and the authors speculated that the high viral load (>10,000 copies/mL) observed in ~50% of individuals at time of emergent drug resistance may indicate that treatment interruption or inconsistent ART adherence occurred. Overall, VF rates remain low across cohorts using DTG + 3TC + ABC in real-world settings.

#### 3.1.2. Dolutegravir/Lamivudine (DTG/3TC) or Dolutegravir/Rilpivirine (DTG/RPV)

Two single-center studies evaluated the effectiveness of switching to DTG-based 2-drug regimens. A retrospective review of data and prescription records for 561 people living with HIV who switched to or continued a DTG-based 2-drug regimen (DTG + 3TC, RPV, or FTC) between January 2015 and October 2021 reported that 6 (1.1%) individuals experienced VF: 4 using a DTG/3TC single-tablet regimen, 1 using a DTG + 3TC multi-tablet regimen, and 1 using a DTG + RPV multi-tablet regimen [42]. One case of VF was attributed to treatment non-adherence. Most individuals (90%) did not have integrase resistance testing performed before starting DTG-based therapy. Treatment-emergent integrase (T66A, G118R, E138K) and reverse transcriptase (M184V, K103N) mutations were detected in 1 individual (0.2%) after VF on a DTG-based regimen (either previous TDF/3TC/DTG regimen or DTG + 3TC multi-tablet regimen at VF) [42]. Another study of a cohort in Spain reported outcomes for 358 people living with HIV switching to DTG + 3TC before August 2019, which included 17 (4.7%) with a pre-existing M184V resistance mutation. Of the 13 (3.6%) who experienced VF at week 144, none had pre-existing M184V and 1 had treatment-emergent INSTI (R263K) and NRTI (M184V) RAMs; additionally, 1 had treatment-emergent M184V when treatment was changed due to VF at Month 24 [39].

The French Dat’AIDS multicenter cohort reported INSTI resistance outcomes in treatment-experienced, virologically suppressed individuals who switched to DTG/RPV (*n* = 799) and DTG + XTC (*n* = 575; *n* = 488 [84.9%] with 3TC) between January 2014 and September 2018. Overall, 464 (33.8%) had a history of prior VF [37]. After a median follow-up of ~20 months, VF was reported in 30 (3.8%) individuals receiving DTG/RPV and 15 (2.6%) receiving DTG + XTC. Of 23 individuals with genotypes available at VF, 2 receiving DTG/RPV had treatment-emergent NNRTI (1 with E138A and L100I; 1 with E138K and K101E) and INSTI RAMs (L74I and N155H, respectively, although polymorphic L741I alone does not reduce DTG susceptibility and N155H alone has minimal effect on DTG susceptibility). The low incidence of treatment-emergent resistance with DTG + 3TC and DTG/RPV is consistent with systematic literature reviews of their use in real-world settings [64,65].

#### 3.1.3. Tenofovir Disoproxil Fumarate/Lamivudine/Dolutegravir (TLD)

Two multicenter studies described the transition from older ART regimens to TLD in low- and middle-income countries (LMICs). In Tanzania in March 2019, eligible children (*n* = 92) and adults (*n* = 45) with high-level viremia (viral load ≥1000 copies/mL) on ART were surveyed for prevalence and patterns of acquired RAMs [62]. Mutations conferring resistance to INSTIs, NNRTIs, and NRTIs were detected in 5.8%, 62.8%, and 44.5% of all individuals, respectively. Among the 82 individuals receiving DTG-based regimens, 4 (4.9%) had at least 1 major INSTI RAM (*n* = 2, E138K; *n* = 2, G118R; *n* = 1, Q148K; *n* = 1, G140A; *n* = 1, T66A; *n* = 1, R263K). The 3 individuals with major INSTI RAMs with reverse transcriptase–protease sequences available also had multiple NNRTI mutations and mutations that conferred resistance to 3TC and TDF. RAMs could not be confirmed as treatment-emergent at VF, as historical or baseline resistance was not available or reported. In 2018, Malawi transitioned 750,000 ART-experienced individuals to TLD in the absence of viral load testing. A prospective observational study of a subset of individuals 1 year after this transition reported that 163/1892 (8.6%) were viremic (HIV-1 RNA ≥50 copies/mL) at baseline; 89 of them were successfully genotyped [41]. At baseline, 42 (47.2%) individuals had dual 3TC and TDF resistance, 11 (12.4%) had resistance to 3TC alone, and none had INSTI resistance. Two individuals (out of 1838 with ≥2 viral load tests, or 0.1%) experienced VF and DTG resistance (R263K and G118R) at month 6 of treatment. Both were viremic and resistant to 3TC and TDF at baseline, suggesting an incidence of INSTI resistance of 4.7% (2/42) among individuals with 3TC and TDF resistance who were potentially receiving only 1 fully active agent (DTG).

A study based on database searches investigated 113 treatment-naive individuals with failure of first-line TLD therapy in Brazil [31]. Seven individuals (6.2%) had major INSTI RAMs at VF; of these individuals, 4 harbored the DTG-specific R263K substitution. In addition, 2 people with R263K also had reverse transcriptase mutations (K70E and M184V). Overall, 13 (11.5%) individuals had RAMs selective for tenofovir and/or 3TC (*n* = 9, M184V; *n* = 4, K70E and M184V) [31]. A single-hospital study in Nigeria in 2021 showed that among 4263 people living with HIV and on a DTG-based regimen for at least 6 months, 281 (6.6%) had a viral load >1000 copies/mL. From this group, resistance testing was completed in 33 individuals on TLD, 1 of whom was known to have vertically acquired HIV and had detectable INSTI RAMs [46]. In addition to multiple INSTI RAMs, including E138K, G118R, T66A, and R263K, this individual showed high-level resistance to NNRTIs and NRTIs. Of individuals with resistance testing, 24 (72.7%) had NNRTI RAMs, 17 (51.5%) had NRTI RAMs, and 4 (12.1%) had PI RAMs.

In LMICs, the risk of emergent resistance may be higher due to infrequent viral load monitoring. A study of 716 people living with HIV who switched to TLD in Mozambique between August 2021 and February 2022 identified VF in 216 individuals and successfully obtained genotypes from 167 [45]. Intermediate- to high-level DTG resistance was observed in 35/167 (21.0%) individuals and occurred more frequently in those with viremia (19.4%) or lack of viral load testing (40.5%) before they switched to TLD. In this study, the INSTI mutations detected were G118R, G140A/C/R/S, Q148H/K/R, N155H, Y143C/H/R, and R263K. Moreover, 10/35 (28.6%) demonstrated resistance to each drug in the regimen [45].

The results from the DTG RESIST study, which includes multiple cohorts that are part of the ART Cohort Collaboration and the International epidemiology Databases to Evaluate AIDS (Europe, North America, South Africa), highlight the importance of genotypic resistance testing, particularly in individuals with prior treatment experience [48]. Among 599 people living with HIV included in the analysis, 86 (14.4%) developed at least 1 major or minor INSTI RAM, and 20 (3.3%) had >1 INSTI RAM. While 563 (94.0%) individuals retained full DTG susceptibility, 17 (2.8%) and 6 (1.0%) showed intermediate- and high-level resistance, respectively. The most frequently detected INSTI RAM was R263K (*n* = 10). Factors from this analysis associated with DTG resistance included DTG monotherapy, DTG + 3TC 2-drug therapy, and NRTI backbone resistance. There was a strong association between DTG and NRTI resistance in sensitivity analyses of individuals with only 3TC and TDF resistance. Furthermore, evaluation of individuals with available historical genotypes suggested that prior resistance to the NRTI backbone was associated with an increased likelihood of treatment-emergent DTG resistance [48].

### 3.2. Risk Factors for Treatment-Emergent Resistance

Our review of INSTI RAMs highlighted several factors associated with resistance development, including suboptimal adherence, DTG monotherapy, lack of viral load testing, prior VF, and extensive treatment experience.

#### 3.2.1. DTG Monotherapy

The efficacy and high barrier to resistance of DTG have prompted investigation into the possibility of its use as monotherapy. Three published reports of 2 cohorts treated with DTG monotherapy describe VF outcomes with resistance testing. These single-center European studies included individuals with extensive prior ART experience and suppressed viremia for at least 6 months before switching to DTG monotherapy. In one study (N = 28), 3 people living with HIV with previous exposure to first-generation INSTIs RAL or EVG experienced VF during 24 weeks of DTG monotherapy. Treatment-emergent DTG resistance (E138K, Q140A, Q148R, *n* = 1; N155H, *n* = 1) was detected in 2 individuals [63]. A subsequent report of this study included 61 people living with HIV who were on DTG monotherapy for a median of 100 weeks, and no additional cases of VF or INSTI resistance were observed [40]. In a separate study of 31 individuals with 24 weeks of follow-up, there was 1 case of VF with DTG resistance (Q148H, G140S) and a minor PI-related mutation (A71V) [49]. Although these real-world studies show a relatively low incidence of INSTI resistance, it is worth noting that DTG monotherapy demonstrated inferiority to combination ART at a 48-week follow-up in a randomized non-inferiority trial, with treatment-emergent INSTI RAMs detected in 3/95 (3.2%) participants receiving DTG monotherapy (S230R, R263K, N155H) [66]. Overall, the risks of monotherapy appear to outweigh any potential benefits.

#### 3.2.2. Prior Virologic Failure

Based on data from a large Italian cohort in the ARCA database, RAMs were evaluated in 107 INSTI-experienced viremic people living with HIV [51]. Of 13 people receiving DTG-based regimens, 1 (7.7%) had an R263K mutation before INSTI treatment; 3 (23.1%) had major treatment-emergent INSTI RAMs at VF (2 each with N155H, G140S, and Q148H; and 1 each had Y143C/H/R and E138A).

A 6-year retrospective study in Germany included 655 (2017–2018), 710 (2019–2020), and 667 (2021–July 2022) samples from individuals with viremia taking second-generation INSTIs [61]. Approximately half of the samples from individuals with historical resistance data had previous NRTI and/or NNRTI RAMs. Emergence of INSTI resistance was rare, with no major INSTI RAMs detected in the 3% of samples with VF. Among individuals using DTG-based regimens (N not reported), new INSTI RAMs developed in 4 individuals at VF (R263K) and were detected in 4 additional individuals at VF without historical resistance results to confirm treatment emergence (R263K, *n* = 2; G118R, *n* = 2; T66I, *n* = 1; E138K, *n* = 1). Furthermore, reverse transcriptase RAMs developed in 2 individuals at VF (K70E, K101E) and were detected in 4 additional individuals at VF (M184V/I, *n* = 4; L210F, *n* = 1; K103R, *n* = 1; and V179I, *n* = 1) without historical context to confirm emergence.

#### 3.2.3. Heavily Treatment-Experienced Populations

In the multicenter COPEDOL study in France, 459 people living with HIV with treatment failure on prior ART were treated with DTG-based regimens for 24 months [38]. Individuals had prior exposure to an average of 7 ART regimens (*n* = 150 [32.7%] with previous INSTI exposure), and of 311 individuals with genotyping analysis, ~25% had only 1 fully active ARV available. In this HTE population, 94 (21.4%) cases of VF were reported in 440 individuals, 5 of whom developed INSTI RAMs; however, a G140A/C/S mutation was detected at study inclusion in 1 of these 5.

First-generation INSTIs (RAL and EVG) have a relatively low barrier to resistance, and people living with HIV with prior exposure to these drugs can develop RAMs that also confer resistance to DTG [12]. In a retrospective multicenter cohort study of 467 individuals who experienced VF while on a DTG-based regimen in France and Italy, 58 (12.4%) had at least 1 major INSTI RAM [35]. INSTI RAMs were more frequent in individuals with first-generation INSTI experience (21.2%; *n* = 46) compared with INSTI-naive individuals (3.9%; *n* = 9). Of the 9 INSTI-naive individuals with INSTI RAMs at VF, 2 had NRTI RAMs (M184V), and 1 had NNRTI RAMs (K103N and E138G). Among RAMs specifically associated with DTG resistance, R263K and G118R had a prevalence of <2%. An Italian PRESTIGIO database analysis of 190 people living with HIV with INSTI resistance from first-generation INSTI exposure reported that, of 142 individuals with available baseline resistance data, 105 (73.9%) had ≥1 major INSTI mutation [36]. Moreover, 48 individuals (25.3%) experienced VF with twice-daily DTG + OBT, 16 of whom had follow-up resistance testing, which showed minimal evolution of mutations. Presence of Q148H/K/N/R and G140A/C/S at baseline plus 1 or more INSTI RAMs was associated with a significantly shorter time to VF. Nevertheless, this study suggests the benefit of DTG + OBT in a population with few treatment options.

A study evaluated resistance in people living with HIV-1 subtype C who experienced VF on DTG and/or RAL-based ART in public health facilities in Botswana, where HIV-1 subtype C is dominant [44]. Plasma samples from 34 individuals were sequenced and 11 (32.4%) had INSTI RAMs; of these, 8 had treatment failure on a DTG-based regimen at resistance sampling. The most frequently detected RAMs among those failing on a DTG-based regimen were E138K (*n* = 5), S147G (*n* = 3), Q148R (*n* = 3), and N155H (*n* = 2; full list in Table 1), with NRTI, NNRTI, and/or PI RAMs also present. In a separate South African cohort of 43 people living with HIV and on third-line ART (*n* = 34 [79.1%] with RAL exposure and *n* = 4 [9.3%] with DTG exposure), 3 (7.0%) individuals demonstrated intermediate- to high-level DTG resistance at VF [52]. One individual had multiple RAMs (T66A, E138K, Y143R, S147G, Q95K, and T97A), while the other 2 had previous exposure to RAL and had mutations conferring cross-resistance to DTG.

Among the congress abstracts and posters reviewed, 3 studies reported a combined prevalence of INSTI resistance of 9.4% (130/1376) among HTE people living with HIV or those with prior exposure to first-generation INSTIs and/or multi-class resistance, with resistance testing available at VF [45,55,57]. Three studies of INSTI-experienced individuals and/or those with prior VF demonstrated a combined prevalence of 15.9% (10/63) for mutations conferring DTG resistance in individuals on DTG-based regimens with resistance testing available at VF, though another study reported resistance in 2 individuals on DTG-based regimens out of 147 total individuals using INSTIs (1.4%) [56,58,60]. These studies together indicate that even in people living with HIV who are HTE, emergence of mutations that confer major resistance to DTG is low compared with resistance to other drug classes.

#### 3.2.4. Suboptimal Adherence

Intermittent treatment adherence can increase the likelihood of mutations developing, as the drug would be present but with insufficient pressure to fully suppress replication [67], but it is challenging to determine rates of adherence among people living with HIV in clinical cohorts. In a Spanish cohort of 307 people living with HIV who were prescribed DTG, 3 individuals discontinued due to VF, 1 of whom developed DTG resistance [59]. In another Spanish cohort of 33 people living with HIV, 1 individual with previous VF on a RAL-based regimen experienced VF with DTG monotherapy; although no INSTI RAMs were detected at VF, the individual developed G118R 24 weeks into the study, which confers resistance to DTG [50]. Investigators from these cohorts attributed DTG resistance selection and/or VF to suboptimal adherence; however, other risk factors such as prior VF with a first-generation INSTI and DTG monotherapy may have additionally contributed to emergent drug resistance in these cases.

In another cohort of 174 people living with HIV with INSTI resistance, 4 out of 5 individuals with DTG resistance mutations were non-adherent to current or previous DTG-based treatment [47]. Detected INSTI resistance mutations were R263K, E157Q, and S230R. In addition to non-adherence, the authors speculated that the presence of NRTI or NNRTI resistance mutations (e.g., M184V and K103N, respectively) may have also contributed to the development of DTG resistance. Another study similarly found infrequent VF (*n* = 13) among a cohort of 358 people living with HIV who switched to DTG/3TC, and only 1 individual (0.3%; 1/358) developed the intermediate-level INSTI resistance mutation R263K [39]. The authors speculated that VF may have occurred due to suboptimal adherence.

Larger analyses have shown similar findings. Among 955 people living with HIV in Ukraine and Poland, multiple INSTI RAMs (E138K, Q148R, and R263K) were detected in 1 individual with suspected suboptimal adherence after intermittent TLD exposure [54]. In a large analysis of 1892 individuals who transitioned to TLD, 0.8% experienced VF with suspected suboptimal adherence, 2 (0.1%) of whom had DTG resistance (R263K and G118R) after 6 months of treatment [41].

### 3.3. Treatment-Emergent Resistance in People Living with HIV-2

A database search of 319 people living with HIV-2 from the Spanish HIV-2 national register identified 10 individuals who had experienced treatment failure on a RAL-based regimen and had an available integrase sequence through December 2015 [53]. Among these, 9 had INSTI RAMs and 5 switched to a DTG-based regimen. After a median duration of 14 months, 3 individuals who had switched to a DTG-based regimen experienced virologic rebound with emergent Q148K/R (*n* = 2) and G118R (*n* = 1) RAMs [53]. This study provides an evaluation of the DTG resistance barrier in the context of HIV-2, for which treatment options are more limited, with baseline INSTI RAMs.

## 4. Discussion

Here, we conducted a comprehensive review of VF and treatment-emergent RAMs among people living with HIV receiving DTG-based regimens in real-world settings, using publications and congress data from 2013 to 2023. Published reports of real-world evidence are consistent with data from DTG clinical trial programs showing that development of mutations conferring resistance to INSTIs in individuals receiving DTG-based 2-drug and 3-drug regimens is infrequent. Risk factors associated with greater likelihood of emergence of DTG resistance include DTG monotherapy, previous VF on first-generation INSTIs, switching of treatment-experienced individuals in the absence of viral load and/or genotypic resistance testing, and suboptimal ART adherence. These factors suggest that despite the effectiveness of DTG-based regimens in many different populations, clinical vigilance regarding patient histories, frequency of viral load testing, and adherence counseling is required for selection of the appropriate ART regimen. Moreover, continued monitoring of emergent resistance is necessary in large real-world cohorts.

Historical or baseline resistance information is not always available in real-world settings at treatment initiation or switch. Randomized controlled trials of a DTG 2-drug regimen for treatment simplification, including TANGO and SALSA, used baseline resistance and prior VF as exclusion criteria [24,68]. In contrast, 2 studies that assessed use of DTG/3TC (STAT, N = 131) and DTG + 3TC (DOLAVI, N = 88) in a test-and-treat setting reported that a relatively high proportion of individuals initiating treatment achieved virologic suppression (76.3% [100/131] and 86.4% [76/88] by Snapshot algorithm, respectively) over 48 weeks [20,69]. Both studies required baseline resistance testing at study entry, but results were not available until week 4 in STAT, and 84.1% of individuals started same-day treatment in DOLAVI. In a retrospective analysis of the TANDEM sub-cohort of treatment-naive adults initiating DTG/3TC (N = 126), high suppression rates (>83%) were observed regardless of test-and-treat status after a median 1.3-year (test-and-treat, *n* = 61) or 1.2-year (not test-and-treat, *n* = 62) follow-up [70]. Though 71.9% of the Spanish REDOLA cohort study (N = 135) initiated DTG/3TC without a baseline resistance test, high virologic suppression rates (intention-to-treat missing = failure analysis, 85.2% [115/135]; per-protocol analysis, 96.6% [115/119]) were reported at week 48. Of note, 1 individual with M184V detected in their baseline resistance test achieved virologic suppression by week 6 [71]. A meta-analysis of people living with HIV who switched to DTG + 3TC reported low estimated proportions with VF based on data from real-world cohorts at weeks 24, 48, and 96 regardless of the presence of pre-switch M184V/I mutations (with M184V/I: 0.01, 0.03, and 0.04, respectively; without M184V/I, 0.00, 0.02, and 0.02, respectively), which were consistent with estimated proportions with VF in participants with historical M184V/I from interventional studies (0.00, 0.00, and 0.00, respectively) [72]. Rates of VF were also infrequent 24 weeks after first-line initiation of DTG + 3TC (*n* = 106) or DTG + TDF/XTC (*n* = 108) without baseline resistance testing in the randomized phase 4 D2ARLING trial, where no RAMs were observed at VF for the 1 participant taking DTG + TDF/XTC who experienced VF [73]. Findings from these studies and low prevalence of pre-treatment INSTI resistance show that DTG-based regimens result in similar treatment outcomes when pre-treatment resistance testing is not available; however, caveats may arise if other risk factors are present (e.g., previous VF).

In general, INSTI mutations were infrequently detected at VF across studies identified in this review, even in cohorts where individuals had previous VF and/or historical mutations. VF rates ranged from 1% to 60% among the 16 lead studies reporting VF outcomes [33,36,37,38,39,40,41,42,43,45,46,49,50,53,58,59] for individuals on DTG-based regimens (excluding 8 lead studies with 100% VF rates due to study design [31,32,35,44,48,52,60,62], i.e., resistance analyses in populations failing treatment); of note, the highest rates (50% and 60%) were observed in cohorts with N ≤ 5 [53,58]. These results are consistent with other real-world studies [69,70,71] reporting low VF rates and/or infrequent INSTI RAM development in people receiving DTG-based treatment and reinforce the high barrier to resistance observed in interventional studies in ART-naive and ART-experienced individuals [14,15,16,17,18,19,20,21,22,23,24,25,27,73]. However, models from a large (N = 669) Italian cohort of suppressed-switch individuals receiving DTG + 3TC showed that previous VF on an INSTI-based regimen was associated with an increased risk of future VF (adjusted hazard ratio, 5.51; 95% CI, 1.15–26.50) [74], and results from the VIKING-3 trial showed that participants with Q148 + ≥2 mutations had 96% lower odds of suppressing within 24 weeks compared with those without Q148 mutations [26]. Thus, although DTG-inclusive regimens are recommended by guidelines for individuals with VF [1,3,6], including with limited INSTI resistance detected (e.g., due to VF with RAL or EVG, as in VIKING-3), it is important to consider an individual’s treatment history.

We also evaluated studies of DTG-based ART regimens for HTE individuals with few treatment options. Since the introduction of INSTIs, the prevalence of HTE people living with HIV has decreased from 7.5% in 2006 to <1% in 2012 through 2017 [75]. Though this decreased prevalence likely reflects the improvement of ART regimens and increased options for treatment over the last several decades, HTE people can still harbor multi-class resistance that could potentially include INSTI RAMs [76]. Higher prevalence of RAMs was observed in specific groups, such as those who failed and had virus with RAMs to first-generation INSTIs. Still, DTG + OBT was effective in treating people with ≥1 INSTI mutation at baseline, and where VF did occur in this population, patterns of RAMs had sometimes not evolved significantly. Higher incidence of treatment-emergent resistance was also noted in studies of smaller cohorts with multidrug-resistant HIV-1 and previous exposure to RAL. This frequently corresponded with detection of certain RAMs, such as Q148H/K/N/R, which was shown in controlled trials to reduce susceptibility to DTG when present with at least 2 other mutations [75]. The DTG resistance mutation R263K was detected in some cases of VF, but frequency was low. As real-world evidence suggests, treatment of this unique group of individuals requires a thorough evaluation of the reasons for prior VF and RAM profiles as well as an understanding of ARV mechanisms of action and pathways to resistance.

Real-world cohort data suggest that intermittent, suboptimal treatment adherence is a relatively common contributing factor to the emergence of mutations conferring resistance to DTG. Adherence is difficult to estimate from cohort studies, with most relying on subjective measurements such as self-reporting and provider evaluations [77]. Even more objective measures such as electronic monitoring and pill counting have challenges, where the latter can generate inaccurate estimates of adherence due to factors such as surplus medication or the assumption that a dose removed from its container is equivalent to an individual taking the medication. In the phase 3 GEMINI-1 and GEMINI-2 studies, a post hoc analysis showed that rates of virologic suppression were lower among those with more suspected missed doses [78]. Importantly, the 1 participant in the GEMINI studies who developed an emergent INSTI RAM (R263R/K) had a previous elevated viral load attributed to treatment non-adherence [19]. Real-world data showed that suboptimal adherence may increase the risk of RAM selection, particularly in individuals with prior VF and INSTI exposure. Factors such as persistent low-level viremia (ranging from “detectable” HIV-1 RNA levels up to <1000 copies/mL) may point toward intermittent ART use. Indeed, analyses from a cohort of 240 adults receiving DTG/3TC indicated a significant association between adherence and odds of achieving and maintaining HIV-1 RNA <50 or <200 copies/mL (*p* < 0.0001) over 681 person-years of follow-up and that <80% adherence (by proportion of days covered) was associated with poorer virologic outcomes [79]. Although suboptimal adherence is generally defined as <80% adherence to treatment [80,81], recent studies have not determined a specific proportion of missed doses to correlate with resistance development, especially as drug resistance mutations are less likely to develop with longer periods of treatment non-adherence due to insufficient drug pressure [67]. While DTG-based regimens have demonstrated “forgiveness” in their ability to maintain suppression despite suboptimal adherence in clinical trials, to avoid the risk of emergent RAMs, emphasis should be placed on patient counseling and implementation of objective measures in addition to self-reporting.

Considering that most recommended regimens are daily single-tablet oral medications for individuals without prior treatment experience or VF, adherence is expected to improve as regimens become more convenient. A systematic literature review of ART adherence in real-world observational study settings reported that in 9/11 publications evaluating the association between number of tablets in the regimen and adherence to treatment, use of single-tablet regimens was associated with significantly higher adherence compared with multi-tablet regimens [82]. Furthermore, 13/18 studies showed that higher adherence was associated with greater virologic suppression. Though these findings support the high barrier to resistance of DTG-based regimens, people living with HIV should be encouraged to maintain optimal adherence (i.e., “every dose, every day”).

In accordance with the Stanford HIV Drug Resistance Database [83,84], we found that in cases of VF on DTG-based therapy with RAM emergence, the common major mutations detected were at residues G118, E138, G140, Q148, and in some cases R263. These mutations were often detected in HTE individuals and those with prior INSTI exposure or lack of sufficient backbone activity. While substitutions at one of these positions alone, such as E138, does not significantly decrease susceptibility to INSTIs, they have a greater effect when detected in combination [85,86]. The concurrent presence of the Q148H/K/R and G140A/C/S mutations, for example, contributes significantly to the reduction of susceptibility to multiple INSTIs, including DTG [87]. Furthermore, mutations outside of the integrase gene that may affect INSTI efficacy have recently been reported, such as those at the 3′PPT [88,89,90]. While these have been detected in vitro from passage experiments using DTG and rarely in individuals taking INSTI-containing regimens, further studies are needed to evaluate their impact and monitor for novel resistance mutations. However, because this review only identified studies that detected and reported integrase mutations, the overall incidence of treatment-emergent INSTI resistance cannot be assessed.

In summary, the use of DTG-based 2- and 3-drug regimens in real-world settings over the last decade has allowed people living with HIV to achieve or maintain virologic suppression with low incidence of emergent resistance. Risk of VF and treatment-emergent resistance to INSTIs are associated with an individual’s prior treatment history, virologic status and baseline genotype, and adherence to ART. Careful treatment selection and frequent viral load and genotypic testing is required for HTE populations.

## Figures and Tables

**Table 1:** Treatment-Emergent Resistance by DTG Regimen.

Publication	DTG Regimen	People on DTG Regimen, N	GRT Results (Historical and/or Baseline), *n*/N (%)	People with Baseline or Historical Mutations, *n*/N (%) [Mutations]	VF Outcomes, *n*/N (%)			
					Total People with VF	GRT Availability at VF	People with Baseline Mutations and VF	People with Emergent Integrase Mutations at VF [Mutations]
DTG Monotherapy								
Rojas et al., 2016 [50]	DTG monotherapy	33	33/33 (100)	16/33 (48) [15V, M41L, E44D, A62V, K65R, D67N, T69D, K70R, L74V, Y115F, V118I, M184V, L210W/S, T215Y/F, K219E/Q, M46I/L, I50L, L63P, A71V, G73S, V77I, L90M, A98G, K101E, K103N, V106A/I, V108I, Y181I/C, G190S]	1/33 (3)	1/1 (100)	1/1 (100)	1/1 (100) [G118R]
Oldenbuettel et al., 2017 [49]	DTG monotherapy	31	NR	NR	1/31 (3)	1/1 (100)	NR	1/1 (100) [Q148H, G140S] ^a^
Tebano et al., 2020 [40]	DTG monotherapy	61	61/61 (100)	3/25 (12) [E138K, G140S, N155H, S147G, L74I] [63] ^b^	3/61 (5)	3/3 (100)	1/3 (33) [63]	3/3 (100) [E138K, G140A, Q148R, E92Q, N155H]
2DR with DTG								
Deschanvres et al., 2022 Dat’AIDS [37]	DTG/RPV or DTG + XTC	1374	At least 6/1374 (<1)	At least 4/1374 (<1) [K103H/N/S/T, E138K, M184V]	45/1374 (3)	23/45 (51)	4/45 (9)	2/45 (4) [N155H, L74I]
Palmier et al., 2023 [39]	DTG/3TC	358	358/358	17/358 (5) [M184V, K103N] ^c^	13/358 (4)	9/13 (69)	1/13 (8)	1/13 (8) [R263K]
Bowman et al., 2023 [42]	DTG/3TC (majority), DTG/RPV, or DTG/FTC	561	56/561 (10)	2/561 (<1) [F121Y, N155H] ^b^	6/561 (1)	5/6 (83)	0	1/6 (17) [T66A, G118R, E138K]
3DR with DTG								
Lepik et al., 2017 [43]	DTG + 3TC + ABC or DTG + TDF/FTC or TDF/3TC	392	392/392 (100) ^d^	NRTI: 31/392 (8) NNRTI: 40/392 (10) PI: 6/392 (2) INSTI: 3/392 (1)	65/392 (17)	NR	NR	3/65 (5) [T66I, R263K]
Schramm et al., 2022 [41]	TLD	1892	89/1892 (5)	53/1892 (3) [M184V/I, K65R, K70E, L74V/I, Y115F, M41L, D67N, K70R, L210W, T215Y/F, K219Q/E]	37/1762 (2)	14/37 (38)	4/37 (11)	2/37 (5) [R263K, G118R]
Diaz et al., 2023 [31]	TLD	113	NR	NR	113/113 (100) ^e,f^	113/113 (100)	NR ^g^	25/113 (22) [M50I/T/M, V151A/I, L101I/V, R263K/R, G140R, G163R, T97A, L74I/M, E157Q, M154I, G118R, E138A, G149A/G, G193E] ^h^
Kamori et al., 2023 [62]	DTG/TDF/3TC	82	NR	NR	82/82 (100) ^e,i^	82/82 (100) ^e,i^	3/82 (4) ^j^	7/82 (9) [Q148K, E138K, G118R, G140A, T66A, R263K, T97A, Q95Q/K] ^h^
Other								
Requena et al., 2017 [53] (HIV-2)	DTG + DRV/r or DTG + ATV/r, plus 2 nucleos(t)ides	5	5/5 (100)	5/5 (100) [Y143G/C, Q91R/Q, E92E/Q, T97A/T, A119T, A153G/S, I84V, N155H]	3/5 (60)	3/3 (100)	3/3 (100)	3/3 (100) [K4R, K14R, V75A, G118R, A119T, V141I, Q148K/R, V150T, V151I, A153S, Q208H, L220F]
Castagna et al., 2018 [36] PRESTIGIO	DTG 50 mg BID + OBT	190	142/190 (75)	NNRTI: 80/142 (56) PI: 77/142 (54) INSTI: 117/142 (82) NRTI: 96/142 (68)	48/190 (25)	16/48 (33) ^k^	16/48 (33)	9/48 (19) [T97A, E138K, L74I, G140S, Q148H, T66I] ^b,k^
Steegen et al., 2019 [52]	Various	4	NR	NR	4/4 (100) ^e^	NR (assumed 100)	NR	1/4 (25) [T66A, E138K, Y143R, S147G, Q95K, T97A] ^h^
Scutari et al., 2020 [51]	Various	13	13/13 (100)	1/13 (8) [R263K]	All regimens: 102/107 (95)	NR	NR	3 [N155H, G140S, Q148H, E138A, T97A, Y143H/C/R] ^l^
Seatla et al., 2021 [44]	Various	24 (7 unknown, DTG- or RAL-based)	NR	NR	DTG: 24/24 (100) ^e^ DTG or RAL: 7/7 (100) ^e^	NR	NR	8/24 (33) [E138E/A/K/T, G140A, Q148R/K, A128T, G118R, S147G, E157Q, N155N/H/D, D232N, T66A] ^h^
Gil et al., 2022 [47]	Various	NR ^m^	NR	NR	NR ^m^	NR ^m^	NR	8/174 (5) [G163R/K, S230R, R263K, E157Q] ^h,n^
Landman et al., 2022 [38] COPEDOL	Various (including monotherapy)	459	NRTI: 349/459 (76) NNRTI: 350/459 (76) PI: 349/459 (76) DTG: 150/459 (33)	NRTI: 179/349 (51) NNRTI: 154/350 (44) PI: 139/349 (40) DTG: 9/150 (6) [V151L, R263K, E92Q, N155H, Q148H/K/R, L741I, G140A/C/S]	94/440 (21)	192/440 (44)	14/440 (3)	5/440 (1) [E138A/K/T, G140A/C/S, T66K + L74M, S153F, E157Q] ^o^
Abdullahi et al., 2023 [46]	Various	4263	NR	NR	281/4263 (7) ^p^	33/281 (12; all DTG + 3TC + TDF)	NR	1/281 (<1) [T66A, G118R, E138K, R263K] ^h^
Armenia et al., 2023 [35]	Various (including monotherapy)	467	NR	NR	467/467 (100) ^e^	467/467 (100) ^e^	NR	Total: 58/467 (12) INSTI-naive: *n* = 9 (2) ^g^ INSTI-experienced: *n* = 46 (10) ^h^ [N155H, R263K, E138A/K/T, S147G, E92A/Q, G140A/C/S, Q148H/R, D232N, T97A, T66A/I, G118R, L74I, V151A/I, E157Q, L74M, G163R, S153F, H51Y, P142T, Y143S, G149A]
Loosli et al., 2023 [48]	Various (including monotherapy)	599	395/599 (66)	NR	599/599 (100) ^e^	NR	NR	86/599 (14) ^h^ INSTI-naive: *n* = 28 (5) ^g^ [T66A/I/K/R, E92G/Q, G118R, E138K, G140E/K/R/S, Y143C, S147G, Q148H/R, N155H, R263K, A49G, H51Y, Q95K, T97A, A128T, P142T, Q146L/K, E157Q, G163K/R/S, S230R, D232N]
Parczewski et al., 2023 [54]	Various	57.06% of 842 (all regimens)	NR	NR	*n* = 3	3/3 (100)	NR	1/3 (33) ^h^ [E138K, Q148R, R263K]
**Congress Abstract**	**DTG Regimen**	**People on DTG Regimen, N**	**GRT Results (Historical and/or Baseline), *n*/N (%)**	**People with Baseline or Historical Mutations, *n*/N (%) [Mutations]**	**VF Outcomes, *n*/N (%)**			
					**Total People** **with VF**	**GRT Availability** **at VF**	**People with Baseline Mutations and VF**	**People with Emergent Integrase Mutations at VF [Mutations]**
2DR/3DR with DTG								
Marcelin et al., EACS 2023 [33]	DTG + 3TC, DTG + RPV, DTG/3TC/ABC	3219	NR	NR	179/3219 (6)	179/179 (100)	NR	3/179 (2) [G140S, Q148H, E92K, N155H]
3DR with DTG								
Bhatt et al., IAC 2023 [45]	TLD	716	NR	NR	216/716 (30)	167/216 (77)	NR	35/167 (21) [G118R, N155H G140S/A/C/R, Q148H/R/K, Y143R/H/C, R263K] ^h^
INSTI-based regimens								
Chieffo et al., EACS 2017 [55]	INSTI-based regimens	40 (all regimens)	NR	EVG: 39/40 (98) ^b^ RAL: 36/40 (90) ^b^ DTG: 8/40 (20) ^b^ [N155H/N, Q148H/Q, G140A/S, T97A, Y143C/R, E138A/K, E92Q, T66I]	All regimens: 12/40 (30) ^q^	NR (assumed 100)	NR	2/12 (17) [NR]
López Brull et al., GeSIDA 2019 [56]	INSTI-based regimens	147 (all regimens)	NR	Naive (all regimens): 4/106 (4) [A128T, E157Q]	Experienced (all regimens): 41/41 (100) ^e^	NR (assumed 100)	NR	6/41 (15) [G118R, R263K] ^c^
Nagel et al., IDWeek 2022 [57]	INSTI-based regimens	1169 (all regimens)	NR	NR	On DTG regimens: 22 All regimens: 102/1169 (9)	On DTG regimens: 22/22 (100)	NR	All INSTI regimens: 58/102 (57) [N155H, E92Q, Q148H/R, S147G, T66I/K, E138A/K/T, G140A/S, R263K, Y143R] ^h,m^
Wiesmann et al., HIV Glasgow 2022 [61]	Second-generation INSTI-based regimens	2032 samples (all regimens)	NR	NR [On DTG regimens: K101R, V106I, V179A/I, M184V, R263K, E138A]	All regimens: 2032/2032 (100) ^e^	2032/2032 (100) ^f^	On DTG regimens: 5 ^r^	On DTG regimens: 4 [R263K] ^r^
Marom et al., EACS 2023 [34]	INSTI-based regimens	209 (DTG-based regimens) 362 (all regimens)	NR (assumed 100)	NR	All regimens: 72/362 (20) ^m^	NR (assumed 100)	NR	All regimens: 25/72 (35) ^h,m^ [R263K, Y143R, G140S, N155H, E92Q, E138K, S147G, Q148R, E157Q, T97A, V151I, L74M, S230R, Q146P ^s^]
Other/Unspecified								
Nithianathan et al., BHIVA 2013 [58]	Unspecified	2	NR	NR	1/2 (50)	1/1 (100)	NR	1/1 (100) [E138K, G140S, Q148H] ^h^
Pulido et al., GeSIDA 2016 [59]	DTG-based regimen	307	NR	NR; *n* = 1 with resistance to RAL	3/307 (1)	NR (at least 1)	1/3 (33)	1/3 (33) [NR; selective for DTG]
Viciana et al., HIV Glasgow 2018 [60]	Unspecified	61	NR	NR	61/61 (100) ^e^	NR (assumed 100)	NR	9/61 (15) [R263K, E138K, Q148H, N155H] ^c,h^
Perry et al., IAC 2023 [32]	DTG- or PI-based regimen	251 (all regimens)	NR	NR	251/251 (100) ^e^	INSTI region: 13/251 (5)	NR	2/251 (1) [E138A/K, G140A, Q148R, R263K] ^h^

ART, antiretroviral therapy; ATV, atazanavir; BHIVA, British HIV Association; BID, twice-daily dosing; 2DR, 2-drug regimen; 3DR, 3-drug regimen; DRV, darunavir; DTG, dolutegravir; EACS, European AIDS Clinical Society; FTC, emtricitabine; GRT, genotypic resistance test; IAC, International AIDS Conference; INSTI, integrase strand transfer inhibitor; NNRTI, non-nucleoside reverse transcriptase inhibitor; NR, not reported; OBT, optimized background therapy; PI, protease inhibitor; PR-RT, protease–reverse transcriptase; r, ritonavir; RAL, raltegravir; RAM, resistance-associated mutation; RPV, rilpivirine; TDF, tenofovir disoproxil fumarate; TLD, tenofovir/3TC/DTG; VF, virologic failure. ^a^ Assumption; individual had no previous documented VF on DTG + FTC/TDF ART. ^b^ Only integrase resistance was evaluated. ^c^ Full results were not reported. ^d^ PR-RT; not all had integrase GRT. ^e^ All individuals had VF per analysis design. ^f^ Of the samples, 1 failed PR-RT PCR amplification. ^g^ Participants were ART-naive and/or INSTI-naive. ^h^ Whether RAMs were pre-existing or emergent could not be determined; full GRT results pre-INSTI or pre-DTG were not explicitly reported. ^i^ Only individuals with viral load ≥1000 copies/mL were analyzed; full cohort (N = 137) sequencing success rates were 99% (integrase) and 98% (PR-RT). ^j^ Authors suggest that 3 individuals had possible historical NRTI resistance. ^k^ Of the 9 individuals who developed emergent INSTI RAMs, 7 had G140 and Q148 mutations at baseline. ^l^ Only individuals who failed INSTI treatment and had emergent resistance were reported. Unknown whether RAM was emergent in 1 individual with T97A at VF. ^m^ Study did not differentiate DTG-specific data from full cohort data. ^n^ *n*/N represents individuals exposed to only DTG/individuals with INSTI RAMs on any regimen. ^o^ Of those studied, 5 individuals developed DTG RAMs; those listed here are RAMs described as being present in more individuals at the end of the study compared with at the start. ^p^ Viral loads >1000 copies/mL; 61/281 (22%) had successful plasma collection. ^q^ Individuals who failed to achieve virologic suppression on INSTI after starting an optimized regimen. ^r^ Here, 4 individuals receiving DTG-based regimens had unspecified historical RAMs but had INSTI RAMs at VF (R263K, *n* = 2; G118R, *n* = 2; T66I, *n* = 1; and E138K, *n* = 1). ^s^ Assumption; reported as a minor mutation of “146qr”.

## Data Availability

No new data were created or analyzed in this study. Data sharing is not applicable to this article.

## Supplementary Materials

The following supporting information can be downloaded at: https://www.mdpi.com/article/10.3390/v15122426/s1, Figure S1: Flow chart of manuscript and abstract selection process; Table S1: PubMed search strategy for manuscripts (25 July 2023); Table S2: Embase search strategy for manuscripts (25 July 2023); Table S3: Cochrane search strategy for manuscripts (25 July 2023).
