# Supplementary material for: A Comprehensive Literature Review of Treatment-Emergent Integrase Resistance with Dolutegravir-Based Regimens in Real-World Settings
Source: Viruses. 2023 Dec 14;15(12):2426. doi: 10.3390/v15122426 (PMC10747437; doi:10.3390/v15122426)
Supplement: Supplementary file 1 [file viruses-15-02426-s001.zip › Figure S1.pdf]

Figure S1. (A) Flow chart of manuscript selection process. 802 studies were identified through a PubMed search performed in July 2023 using the search string defined in Table S1. Of 698 included for screening, 58 studies met inclusion criteria, containing data on DTG resistance and mutations emerging under DTG selection pressure in case studies and retrospective analyses. An additional 2 publications were added post hoc after a manual search of screened abstract titles from the abstract selection process. **(B)** Flow chart of abstract selection process. 982 abstracts were identified through Embase, Cochrane, and manual congress searches performed in July 2023 using the search strings defined in Tables S2 and S3. Of those included for screening, 38 studies met inclusion criteria, containing data on DTG resistance and mutations emerging under DTG selection pressure in case studies and retrospective analyses. A post hoc manual search was performed on October 20, 2023, to capture IDWeek and EACS 2023 congress materials (n=6), 3 of which met screening inclusion criteria. Manual congress searches were conducted for the following: Asian Conference on Hepatitis and AIDS, Alliance Francophone de Acteurs de santé contre le VIH, International AIDS Society/International AIDS Conference, Australasian HIV & AIDS Conference, National Conference of AIDS Society of India, British Association for Sexual Health and HIV, British HIV Association Spring Conference, Canadian Conference on HIV/AIDS Research, Conference on Retroviruses and Opportunistic Infections, EACS, Grupo de Estudio del SIDA-SEIMC, HIV Drug Therapy Glasgow, HIV & Hepatitis in the Americas, The HIV Netherlands Australia Thailand Research Collaboration, International Conference on Antiviral Research, International Conference on AIDS and STIs in Africa, International Congress on Infectious Diseases, IDWeek, Professional Society for Health Economics and Outcomes Research (ISPOR), Japanese Society for AIDS Research, Kenya Association of Physicians, and Société Française de Lutte contre le Sida. DTG, dolutegravir; EACS, European AIDS Clinical Society; INSTI, integrase strand transfer inhibitor; STI, sexually transmitted infection. [a]Non-relevant study types included interventional/randomized controlled trials, in vitro/animal/other preclinical, general reviews, opinion/comments/perspective pieces,

news/guidelines/expert opinion, congress summaries/books, cost-effectiveness/modeling studies, pediatric studies, publication duplication, non-disease/product focused, assay development (technical assay development studies), and erratum.

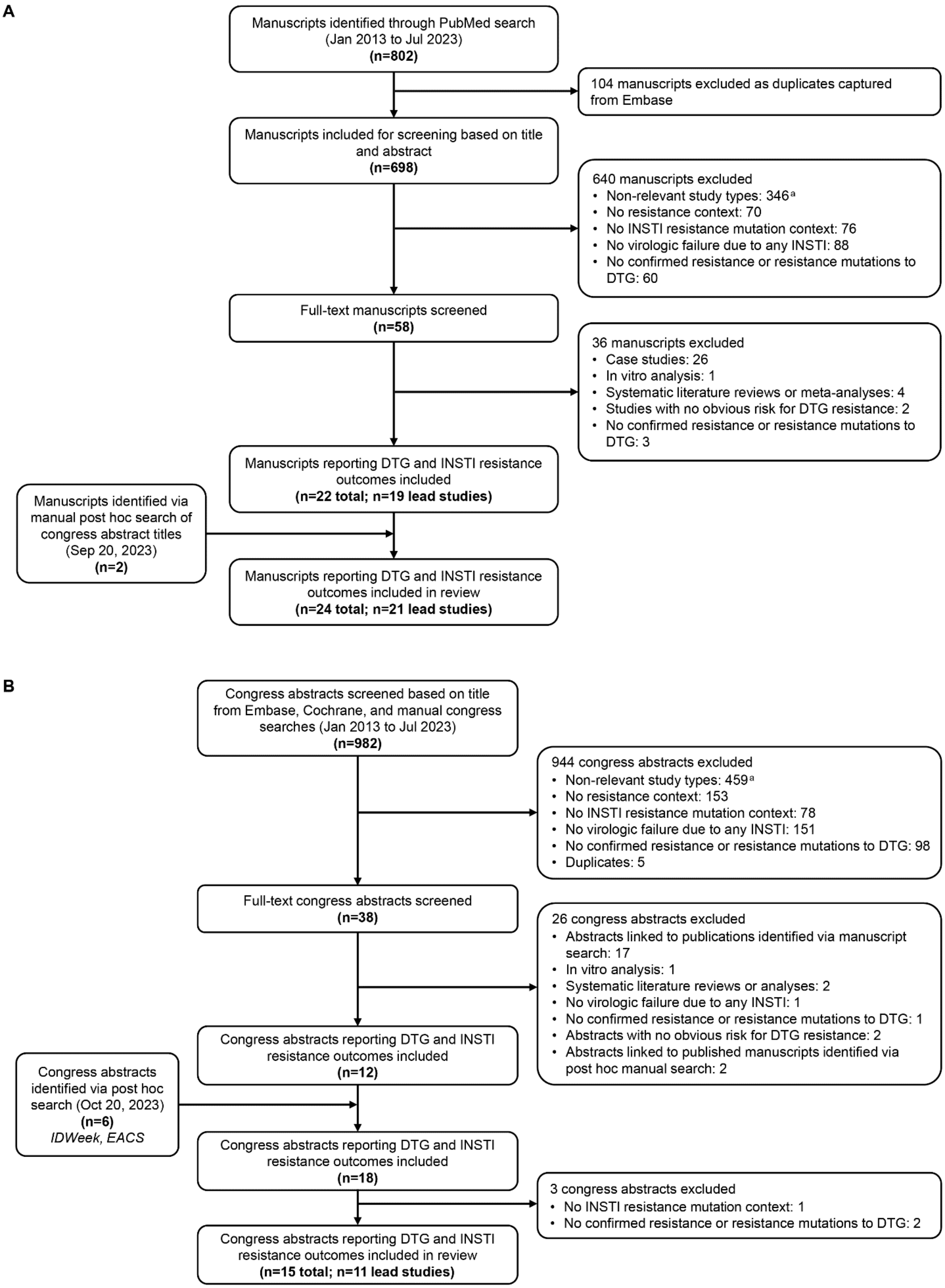
A
Manuscripts identified through PubMed search (Jan 2013 to Jul 2023) (n=802)
104 manuscripts excluded as duplicates captured from Embase
Manuscripts included for screening based on title and abstract (n=698)
640 manuscripts excluded
• Non-relevant study types: 346[a]
• No resistance context: 70
• No INSTI resistance mutation context: 76
• No virologic failure due to any INSTI: 88
• No confirmed resistance or resistance mutations to DTG: 60
Full-text manuscripts screened (n=58)
36 manuscripts excluded
• Case studies: 26
• In vitro analysis: 1
• Systematic literature reviews or meta-analyses: 4
• Studies with no obvious risk for DTG resistance: 2
• No confirmed resistance or resistance mutations to DTG: 3
Manuscripts reporting DTG and INSTI resistance outcomes included (n=22 total; n=19 lead studies)
Manuscripts identified via manual post hoc search of congress abstract titles (Sep 20, 2023) (n=2)
Manuscripts reporting DTG and INSTI resistance outcomes included in review (n=24 total; n=21 lead studies)
B
Congress abstracts screened based on title from Embase, Cochrane, and manual congress searches (Jan 2013 to Jul 2023) (n=982)
944 congress abstracts excluded
• Non-relevant study types: 459[a]
• No resistance context: 153
• No INSTI resistance mutation context: 78
• No virologic failure due to any INSTI: 151
• No confirmed resistance or resistance mutations to DTG: 98
• Duplicates: 5
Full-text congress abstracts screened (n=38)
26 congress abstracts excluded
• Abstracts linked to publications identified via manuscript search: 17
• In vitro analysis: 1
• Systematic literature reviews or analyses: 2
• No virologic failure due to any INSTI: 1
• No confirmed resistance or resistance mutations to DTG: 1
• Abstracts with no obvious risk for DTG resistance: 2
• Abstracts linked to published manuscripts identified via post hoc manual search: 2
Congress abstracts reporting DTG and INSTI resistance outcomes included (n=12)
Congress abstracts identified via post hoc search (Oct 20, 2023) (n=6) IDWeek, EACS
Congress abstracts reporting DTG and INSTI resistance outcomes included (n=18)
3 congress abstracts excluded
• No INSTI resistance mutation context: 1
• No confirmed resistance or resistance mutations to DTG: 2
Congress abstracts reporting DTG and INSTI resistance outcomes included in review (n=15 total; n=11 lead studies)
