# Supplementary material for: A Comprehensive Literature Review of Treatment-Emergent Integrase Resistance with Dolutegravir-Based Regimens in Real-World Settings
Source: Viruses. 2023 Dec 14;15(12):2426. doi: 10.3390/v15122426 (PMC10747437; doi:10.3390/v15122426)
Supplement: Supplementary file 1 [file viruses-15-02426-s001.zip › Table S1.pdf]

Table S1. PubMed Search Strategy for Manuscripts (July 25, 2023)

Search	Query	Results
#13	Search: #12 NOT ("Review"[Publication Type] OR "Editorial"[Publication Type] OR "biography"[Publication Type] OR "comment"[Publication Type] OR "directory"[Publication Type] OR "festschrift"[Publication Type] OR "interview"[Publication Type] OR "legislation"[Publication Type] OR "news"[Publication Type] OR "newspaper article"[Publication Type] OR "patient education handout"[Publication Type] OR "consensus development conference"[Publication Type] OR "consensus development conference, nih"[Publication Type] OR "practice guideline"[Publication Type]) Filters: from 2013 - 2023 Sort by: Most Recent	633
#12	Search: #8 AND #11 Sort by: Most Recent	813
#11	Search: #9 OR #10 Sort by: Most Recent	2,268,176
#10	Search: "3 PPT"[all] OR "A49G"[all] OR "A539V"[all] OR "A556T"[all] OR "CRF14"[all] OR "D232N"[all] OR "E138A"[all] OR "E138K"[all] OR "E138T"[all] OR "E147Q2"[all] OR "E157Q"[all] OR "E92G"[all] OR "E92Q"[all] OR "E92V"[all] OR "F121Y"[all] OR "G118R"[all] OR "G140A"[all] OR "G140C"[all] OR "G140R"[all] OR "G140S"[all] OR "G149A"[all] OR "G163E"[all] OR "G163K"[all] OR "G163R"[all] OR "G19S"[all] OR "H51Y"[all] OR "INRAMS"[all] OR "K65R"[all] OR "K70E"[all] OR "L101I"[all] OR "L74F"[all] OR "L74I"[all] OR "L74M"[all] OR "M184V"[all] OR "M50I"[all] OR "M50V"[all] OR "N155D"[all] OR "N155H"[all] OR "N155S"[all] OR "N155T"[all] OR "P145S"[all] OR "polypurine tract"[all] OR "Q146P"[all] OR "Q148H"[all] OR "Q148K"[all] OR "Q148N"[all] OR "Q148R"[all] OR "Q95K"[all] OR "R263K"[all] OR "S147G"[all] OR "S153A"[all] OR "S153F"[all] OR "S153Y"[all] OR "S230R"[all] OR "T124A"[all] OR "T661"[all] OR "T66A"[all] OR "T66I"[all] OR "T66K"[all] OR "T97A"[all] OR "V151A"[all] OR "V151I"[all] OR "V151L"[all] OR "Y143A"[all] OR "Y143C"[all] OR "Y143G"[all] OR "Y143H"[all] OR "Y143K"[all] OR "Y143R"[all] OR "Y143S"[all] Sort by: Most Recent	2551
#9	Search: "Drug Resistance, Viral"[Mesh] OR "resist*" OR "virological failure"[all] OR "viral failure"[all] OR "incomplete virologic response"[all] OR "incomplete viral response"[all] OR "mutation"[all] OR "mutations"[all] Sort by: Most Recent	2,267,731
#8	Search: #6 AND #7 Sort by: Most Recent	1837
#7	Search: "dolutegravir" OR "dolutegravir sodium" OR "dolutegravir sodium monohydrate" OR "gsk572" OR "gsk 572" OR "gsk 1349572" OR "gsk1349572" OR "gsk 1349572a" OR "gsk1349572a" OR "s gsk 1349572" OR "s gsk1349572" OR "s1349572" OR "s 1349572" OR "s349572" OR "s 349572" OR "dtg"[tiab] OR "tivicay" OR "juluca" OR "triumeq" OR "dovato" Sort by: Most Recent	4019
#6	Search: #1 OR #2 OR #3 OR #4 OR #5 Sort by: Most Recent	850,869
#5	Search: "plwh"[tiab] OR aids[tiab] OR ("acquired immun*" AND "deficiency virus") OR ("acquir*" AND ("immun*" OR "immunodeficiency" OR "immunedeficiency" OR "virus" OR "infection*")) Sort by: Most Recent	338,061
#4	Search: "Acquired Immunodeficiency Syndrome"[Mesh] Sort by: Most Recent	78,606

#3	Search: hiv[tiab] OR "hiv 1"[tiab] OR "hiv 2"[tiab] OR hiv1[tiab] OR hiv2[tiab] OR "hiv i"[tiab] OR "hiv ii"[tiab] OR "hiv infection"[tiab] OR "hiv infect*"[tiab] Sort by: Most Recent	356,198
#2	Search: "human immunodeficiency virus" OR "human immunedeficiency virus" OR ("human" AND ("immun*" OR "immunodeficiency" OR "immunedeficiency") AND ("virus" OR "infection*")) OR ("human immun*" AND "deficiency virus") Sort by: Most Recent	388,953
#1	Search: "HIV"[Mesh] Sort by: Most Recent	107,586

Results (633) exported and screened (search #13).
