# Supplementary material for: A Comprehensive Literature Review of Treatment-Emergent Integrase Resistance with Dolutegravir-Based Regimens in Real-World Settings
Source: Viruses. 2023 Dec 14;15(12):2426. doi: 10.3390/v15122426 (PMC10747437; doi:10.3390/v15122426)
Supplement: Supplementary file 1 [file viruses-15-02426-s001.zip › Table S2.pdf]

Table S2. Embase Search Strategy for Manuscripts (July 25, 2023)

Search	Query	Results
#20	#18 NOT #19	482
#19	#18 AND [medline]/lim	488
#18	#17 AND [embase]/lim	970
#17	#16 AND [2013-2023]/py	991
#16	#15 NOT ('review':de OR 'editorial':de OR 'addresses':de OR 'biography':de OR 'comment':de OR 'directory':de OR 'festschrift':de OR 'interview':de OR 'legislation':de OR 'news':de OR 'newspaper article':de OR 'patient education handout':de OR 'consensus development conference':de OR 'consensus development conference, nih':de OR 'practice guideline':de)	1022
#15	#14 NOT ('animal'/exp NOT 'human'/exp)	1293
#14	#12 AND #13	1301
#13	'cohort analysis':ab,ti OR cohort:ab,ti OR 'follow up':ab,ti OR followup:ab,ti OR 'clinical article':ab,ti OR 'non interventional':ab,ti OR 'noninterventional':ab,ti OR effectiveness:ab,ti OR register:ab,ti OR regist*:ab,ti OR database:ab,ti OR 'data base':ab,ti OR 'real world':ab,ti OR 'real life':ab,ti OR 'health record':ab,ti OR 'health care record':ab,ti OR 'healthcare record':ab,ti OR 'health records':ab,ti OR 'health care records':ab,ti OR 'healthcare records':ab,ti OR 'medical record':ab,ti OR 'medical records':ab,ti OR questionnaire:ab,ti OR survey*:ab,ti OR ((medical:ab,ti OR insurance:ab,ti) AND (claim:ab,ti OR claims:ab,ti)) OR 'billing data':ab,ti OR ((pragmatic:ab,ti OR observation*:ab,ti OR prospective:ab,ti OR retrospective:ab,ti OR 'longitudinal':ab,ti OR 'cross sectional':ab,ti OR clinical:ab,ti) AND (trial:ab,ti OR trials:ab,ti OR study:ab,ti OR studies:ab,ti)) OR (case:ab,ti AND (study:ab,ti OR studies;ab,ti OR series:ab,ti OR large:ab,ti OR control*:ab,ti)) OR ((treatment:ab,ti OR prescrib*:ab,ti OR prescription*:ab,ti) AND (practice*:ab,ti OR practise*:ab,ti OR pattern*:ab,ti))	10,698,751
#12	#8 AND #11	2271
#11	#9 OR #10	3,110,380
#10	3 ppt' OR 'a49g' OR 'a539v' OR 'a556t' OR 'crf14' OR 'd232n' OR 'e138a' OR 'e138k' OR 'e138t' OR 'e147q2' OR 'e157q' OR 'e92g' OR 'e92q' OR 'e92v' OR 'f121y' OR 'g118r' OR 'g140a' OR 'g140c' OR 'g140r' OR 'g140s' OR 'g149a' OR 'g163e' OR 'g163k' OR 'g163r' OR 'g19s' OR 'h51y' OR 'inrams' OR 'k65r' OR 'k70e' OR 'l101i' OR 'l74f' OR 'l74i' OR 'l74m' OR 'm184v' OR 'm50i' OR 'm50v' OR 'n155d' OR 'n155h' OR 'n155s' OR 'n155t' OR 'p145s' OR 'polypurine tract' OR 'q146p' OR 'q148h' OR 'q148k' OR 'q148n' OR 'q148r' OR 'q95k' OR 'r263k' OR 's147g' OR 's153a' OR 's153f' OR 's153y' OR 's230r' OR 't124a' OR 't661' OR 't66a' OR 't66i' OR 't66k' OR 't97a' OR 'v151a' OR 'v151i' OR 'v151l' OR 'y143a' OR 'y143c' OR 'y143g' OR 'y143h' OR 'y143k' OR 'y143r' OR 'y143s'	3694
#9	antiviral resistance'/exp OR 'resist*' OR 'virological failure' OR 'viral failure' OR 'incomplete virologic response' OR 'incomplete viral response' OR 'mutation' OR 'mutations'	3,109,818
#8	#6 AND #7	6010

#7	dolutegravir' OR 'dolutegravir sodium' OR 'dolutegravir sodium monohydrate' OR 'gsk572' OR 'gsk 572' OR 'gsk 1349572' OR 'gsk1349572' OR 'gsk 1349572a' OR 'gsk1349572a' OR 's gsk 1349572' OR 's gsk1349572' OR 's1349572' OR 's 1349572' OR 's349572' OR 's 349572' OR 'dtg':ab,ti OR 'tivicay' OR 'juluca' OR 'triumeq' OR 'dovato'	8588
#6	#1 OR #2 OR #3 OR #4 OR #5	2,022,314
#5	plwh':ab,ti OR aids:ab,ti OR ('acquired immun*' AND 'deficiency virus') OR (acquir* AND (immun* OR immunodeficiency OR immunedeficiency OR virus OR infection*))	445,246
#4	acquired immune deficiency syndrome'/exp	559,029
#3	hiv:ab,ti OR 'hiv 1':ab,ti OR 'hiv 2':ab,ti OR hiv1:ab,ti OR hiv2:ab,ti OR 'hiv i':ab,ti OR 'hiv ii':ab,ti OR 'hiv infection':ab,ti OR 'hiv infect*':ab,ti	455,391
#2	human immunodeficiency virus' OR 'human immunedeficiency virus' OR (human AND (immun* OR immunodeficiency OR immunedeficiency) AND (virus OR infection*)) OR ('human immun*' AND 'deficiency virus')	1,434,094
#1	'human immunodeficiency virus infection'/exp OR 'human immunodeficiency virus infection'	804,964

Results (482; search #20) exported, de-duped, and screened (108 manuscripts; 373 congress abstracts and 1 congress review excluded).
