# Supplementary material for: A Comprehensive Literature Review of Treatment-Emergent Integrase Resistance with Dolutegravir-Based Regimens in Real-World Settings
Source: Viruses. 2023 Dec 14;15(12):2426. doi: 10.3390/v15122426 (PMC10747437; doi:10.3390/v15122426)
Supplement: Supplementary file 1 [file viruses-15-02426-s001.zip › Table S3.pdf]

**Table S3.** Cochrane Search Strategy for Manuscripts (July 25, 2023)

| Search | Query | Results |
|---|---|---|
| #13 | #8 AND #12 with Publication Year from 2013 to 2023, in Trials | 182 |
| #12 | #9 OR #10 OR #11 | 18,295 |
| #11 | "3 PPT" OR "A49G" OR "A539V" OR "A556T" OR "CRF14" OR "D232N" OR "E138A" OR "E138K" OR "E138T" OR "E147Q2" OR "E157Q" OR "E92G" OR "E92Q" OR "E92V" OR "F121Y" OR "G118R" OR "G140A" OR "G140C" OR "G140R" OR "G140S" OR "G149A" OR "G163E" OR "G163K" OR "G163R" OR "G19S" OR "H51Y" OR "INRAMS" OR "K65R" OR "K70E" OR "L101I" OR "L74F" OR "L74I" OR "L74M" OR "M184V" OR "M50I" OR "M50V" OR "N155D" OR "N155H" OR "N155S" OR "N155T" OR "P145S" OR "polypurine tract" OR "Q146P" OR "Q148H" OR "Q148K" OR "Q148N" OR "Q148R" OR "Q95K" OR "R263K" OR "S147G" OR "S153A" OR "S153F" OR "S153Y" OR "S230R" OR "T124A" OR "T661" OR "T66A" OR "T66I" OR "T66K" OR "T97A" OR "V151A" OR "V151I" OR "V151L" OR "Y143A" OR "Y143C" OR "Y143G" OR "Y143H" OR "Y143K" OR "Y143R" OR "Y143S" | 252 |
| #10 | "resist*" OR "virological failure" OR "viral failure" OR "incomplete virologic response" OR "incomplete viral response" OR "mutation" OR "mutations" | 17,921 |
| #9 | MeSH descriptor: [Drug Resistance, Viral] explode all trees | 793 |
| #8 | #6 AND #7 | 815 |
| #7 | "dolutegravir" OR "dolutegravir sodium" OR "dolutegravir sodium monohydrate" OR "gsk572" OR "gsk 572" OR "gsk 1349572" OR "gsk1349572" OR "gsk 1349572a" OR "gsk1349572a" OR "s gsk 1349572" OR "s gsk1349572" OR "s1349572" OR "s 1349572" OR "s349572" OR "s 349572" OR "dtg":ab,ti OR "tivicay" OR "juluca" OR "triumeq" OR "dovato" | 973 |
| #6 | #1 OR #2 OR #3 OR #4 OR #5 | 36,857 |
| #5 | "plwh":ab,ti OR aids:ab,ti OR ("acquired immun*" AND "deficiency virus") OR ("acquir*" AND ("immun*" OR "immunodeficiency" OR "immunedeficiency" OR "virus" OR "infection*")) | 10,424 |
| #4 | MeSH descriptor: [Acquired Immunodeficiency Syndrome] explode all trees | 2267 |
| #3 | hiv:ab,ti OR “hiv 1”:ab,ti OR “hiv 2”:ab,ti OR hiv1:ab,ti OR hiv2:ab,ti OR “hiv i”:ab,ti OR “hiv ii”:ab,ti OR “hiv infection”:ab,ti OR “hiv infect*”:ab,ti | 29,001 |
| #2 | "human immunodeficiency virus" OR "human immunedeficiency virus" OR ("human" AND ("immun*" OR "immunodeficiency" OR "immunedeficiency") AND ("virus" OR "infection*")) OR ("human immun*" AND "deficiency virus") | 15,043 |
| #1 | MeSH descriptor: [HIV] explode all trees | 3750 |

Results (182; search #13) exported, de-duped, and screened (61 articles; 81 congress abstracts and 40 trial overviews excluded).
